# Finding, understanding, and acting on CPR orders: a communication analysis of POLST forms across 51 jurisdictions

**DOI:** 10.3389/fpubh.2026.1979970

**Published:** 2026-10-23

**Authors:** Toni P. Miles, Sydney Catron, Michenzi Brown, Antionette Lavender, Rana Bayakly

**Affiliations:** 1 College of Public Health, University of Georgia, Athens, GA, United States; 2 Spelman College, Atlanta, GA, United States; 3 Georgia Department of Public Health, Epidemiology Division, Atlanta, GA, United States

**Keywords:** communications analysis, end of life, find understand act framework, physicians orders for life sustaining treatment, POLST, portable medical orders, cardiopulmonary resuscitation, CPR

## Abstract

This descriptive communication analysis examines the cardiopulmonary resuscitation (CPR) section of physician/portable orders for life-sustaining treatment (POLST) forms across 51 U.S. Jurisdictions. It focuses on the printable version as encountered by a responder who must locate the CPR order, interpret the physiologic condition that activates it, and determine the authorized response. Printable POLST delivers medical orders on a single 8.5 by 11 inch page. Within this limited space, jurisdictions communicate these orders with slightly different strategies. This study applies a find-understand-act framework to characterize these tasks. Find assesses the visual characteristics of the CPR decision box. Understand examines the relationship between the physiologic trigger, the affirmative or negative treatment choice, and any interpretive burden created by cross-references or adjacent instructions. Act examines language directing response to CPR choice. The prominence of the CPR box and its textual density are two distinct characteristics. Forms anchored the CPR decision to a common physiologic state. However, trigger wording was not linguistically uniform. A yes-CPR response directed initiation of resuscitative actions. A no-CPR response was dominated by instructions not to initiate resuscitation. This option contained few words describing clinical care after a no CPR decision was implemented. This action-language gap is a communication boundary requiring further study. It also presents a challenge to the concept of portable medical orders. Our findings do not rank forms or establish a preferred design; rather, they describe how POLST forms make a time-sensitive CPR decision visible, interpretable, and actionable.

## Introduction

The Physician/Portable Orders for Life-Sustaining Treatment (POLST) documents treatment options for people who have a life-limiting illness, advanced frailty, or both. Completion of a POLST is voluntary. Healthy adults are not eligible for a POLST (1–3). POLST was initially developed in 1991 after clinicians and ethicists recognized that treatment preferences of seriously ill patients were not consistently available nor honored during transitions and emergencies (1). In 2025, the National POLST Collaborative revised the title to emphasize POLST as “portable medical orders,” reflecting a scope that extends beyond CPR to other treatment decisions and goals of care. Between 1991 and 2025, jurisdictions developed individual forms with multiple names, including POLST, Medical Orders for Life-Sustaining Treatment (MOLST), Portable Orders for Sustaining Treatment (POST), Medical Orders for Sustaining Treatment (MOST), and related variants. The National POLST Collaborative also notes that local laws, regulations, and approved forms differ even when programs share a common conceptual model. Typically, clinicians and patients discussing end-of-life treatment options use the Portable Orders form approved in their jurisdiction.

A completed POLST documents treatment choices that have been discussed and translated into medical orders. Signatures of the patient or their spokesperson and the provider indicate agreement with the selected options. This distinguishes POLST from an advance directive (AD). An AD is a broader legal document available to adults who wish to name a proxy and state future health care preferences; POLST converts treatment preferences for persons with serious illness or advanced frailty into orders that clinicians can implement. For patient-centered care near the end of life, those orders carry the expectation that the selected treatment plan will be followed.

Clinical care near the end of life is initiated in response to changing physiologic conditions. Serious illness and advanced frailty are expressed through signs and symptoms that require recognition, interpretation, and treatment. Symptoms associated with the last days of life include anorexia, drowsiness, fatigue, poor wellbeing, and dyspnea, and these may intensify as death approaches (2, 4). The treatment response to these clinical triggers may include comfort-focused care, selective intervention, hospitalization, or resuscitation, depending on the patient’s condition and documented preferences. POLST helps translate previously discussed treatment preferences into medical orders that can guide those responses. Its greatest benefit may lie upstream of the acute care setting, including prehospital settings and nursing homes, where it can interrupt clinical momentum toward unwanted treatment and help align care with documented wishes (3).

Health communication research provides guidance on practices to messaging of medical information. Health literacy in the context of POLST is not limited to reading ability; it also includes understanding and acting on medical information. Both patients and providers can face health literacy challenges when forms are dense, use inconsistent terminology, or lack clear guidance (5–7). Communication research has identified lack of clear language, insufficient white space, and inconsistent typographical organization as potential barriers to literacy. Scholarship examining jurisdictional POLST forms specifically as communication tools remains limited (6). For providers who must implement a documented treatment choice, the communication structure of the form is therefore clinically relevant.

POLST forms are single-page documents that can be printed on an 8.5 by 11 inch page. Within this limited space, the CPR decision appears alongside patient-identification information, instructions, and orders addressing other aspects of care. Figure 1 shows the upper portion of the 2025 National POLST form, cropped to display the CPR decision field and the information encountered before it. The figure provides a visual reference for the communication environment examined in this analysis. On the form created by the National POLST Collaborative, the CPR decision occupies approximately 14% of the upper visual field. Across other jurisdictional forms, both the amount of space devoted to the CPR decision box and the amount of information surrounding it vary.

**Figure 1:**
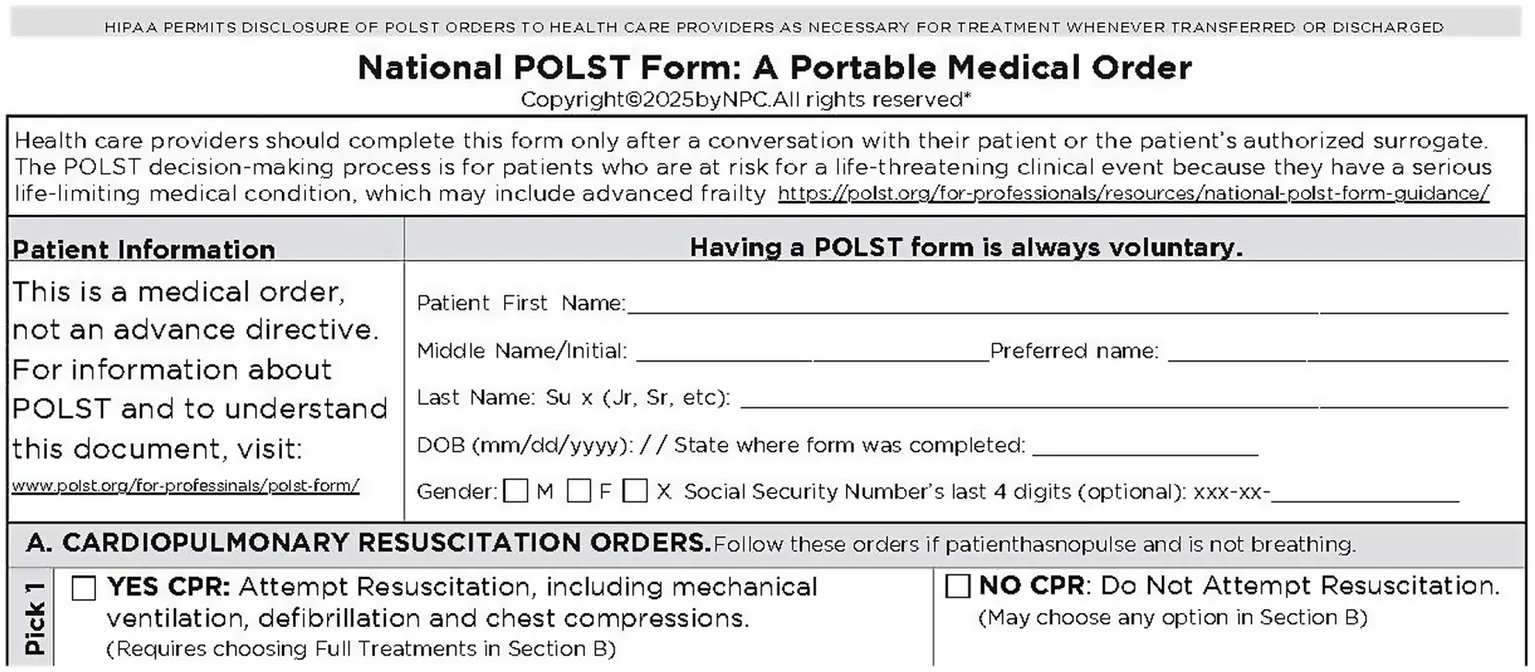
National POLST form—upper field including title, instructions, and CPR decision box. Partial screenshot of the National POLST Form reprinted with permission - Copyright © May 2025 by NPC, https://polst.org/wp-content/uploads/2026/09/National_POLST-Form-9.24.26.pdf.pdf.

The first category of orders in almost all jurisdictions is a directed response to cardiopulmonary resuscitation (CPR). CPR provides a useful analytic case because the decision is time-sensitive and is usually encountered early on the form. In this analysis, the forms are reviewed as communication tools rather than legal instruments. By applying communication science to POLST forms from 51 U.S. jurisdictions, we examine the same clinical decision—yes or no CPR—across differing form structures. The analysis is descriptive rather than evaluative; forms are not ranked and no layout is assumed to be optimal.

To organize the evaluation, we use a Find–Understand–Act conceptual framework. Find asks whether a responder can readily locate the CPR decision within the surrounding information. Understand begins after the decision has been found and asks whether the responder can identify the physiologic condition that activates the order and correctly associate that condition with the authorized treatment choice. Act asks whether the form communicates what response should follow once the selected order is identified and understood. In this way, visual design, clinical trigger language, treatment-choice wording, and responder-directed actions are examined as related but distinct communication tasks. By examining how the CPR decision box is made visible, interpretable, and actionable, this analysis describes the communication work that POLST forms are presently being asked to perform across institutions and settings.

## Methods

We assembled publicly available POLST and POLST-like forms used across 51 U.S. jurisdictions. The National POLST form developed by the National POLST Collaborative was retained as a reference form rather than treated as a jurisdictional observation. This analysis is limited to publicly available printable POLST forms. Electronic or digitally rendered POLST formats, registry interfaces, and electronic health record displays were not evaluated.

The analytic unit was the portion of the page containing the cardiopulmonary resuscitation decision and the material appearing above and immediately adjacent to that field. The analysis focused on communication relevant to an emergency responder who must locate the CPR order, interpret the clinical trigger and treatment choice, and determine the action required. Forms were reviewed as communication documents rather than as legal instruments. Broader treatment-plan sections were examined when explicitly linked to the CPR decision, but the principal analysis is limited to the CPR field and the portion of the form encountered before or with that decision. The analysis is organized around three sequential communication tasks: Find, Understand, and Act. This framework characterizes communication features and does not rank forms or infer responder performance.

Find assesses the visual prominence and accessibility of the CPR decision. For each form, usable page width, upper-field height, and CPR-field height were measured in inches. The upper field extended from the first printed content on page one through the bottom of the CPR decision field. Area measures were calculated from usable width and field height. Derived measures included upper-field area, CPR-field area, and the proportion of the upper-field area occupied by the CPR field. Word counts were recorded separately for the upper field and CPR field, and word density was calculated as words per square inch for each area. The team elected not to capture font size, text spacing, and other aspects of typography because variation across jurisdictions did not lend itself to a systematic measurement approach. Measurements were performed on electronic copies of the printable forms using Adobe measurement tools. Selected forms were measured by two individuals to assess consistency, and discrepant measurements or word counts were adjudicated during review by the full team.

Figure 2 is a scatterplot of two measures—word density within the CPR box (i.e., accessibility) versus CPR field as a portion of the entire upper field (i.e., prominence). The data from this analysis are provided in the Supplementary File. The analysis is descriptive. Continuous visual measures were summarized using means, medians, ranges, and quartiles. Figure 2 examines CPR-field word density in relation to the proportion of upper-field area devoted to the CPR decision. Dashed lines indicate medians among displayed forms. Three extreme forms were omitted from the publication-facing scatterplot to preserve visibility of the central distribution but were retained in descriptive analyses and discussed anonymously. Jurisdiction names were retained in the analytic dataset for verification and adjudication but were not used in publication-facing figures. Oregon was excluded from replicated publication figures because of restrictions on reproduction of the form. A regional/system form retained for provenance review was not treated as a jurisdictional observation.

**Figure 2:**
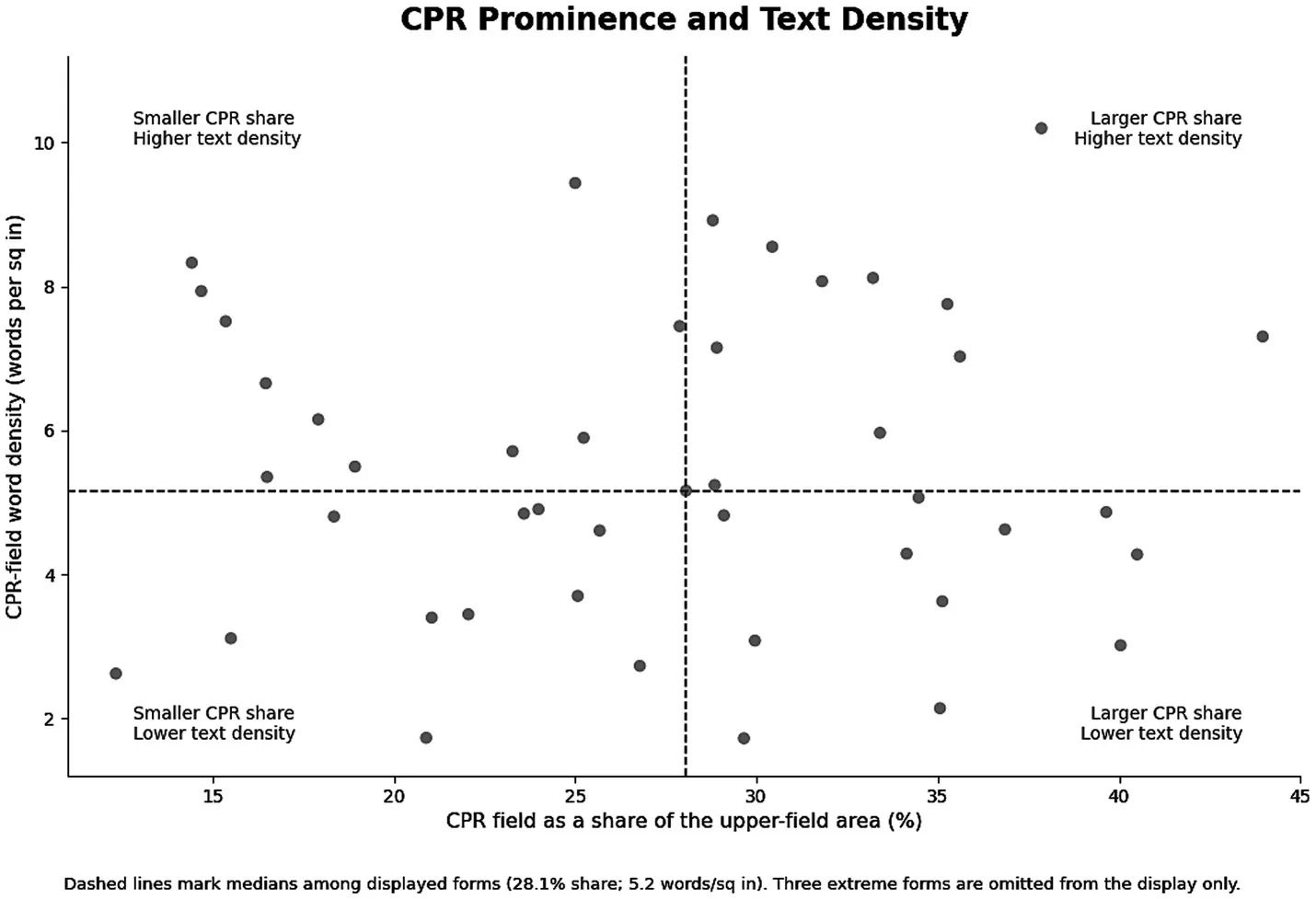
CPR prominence and text density. CPR-field share of the upper visual field is plotted against CPR-field word density. Dashed lines indicate medians among displayed forms. Three extreme forms are omitted from the graphical display only and retained in descriptive analyses.

Understand assesses whether the CPR field communicates a clinically interpretable relationship between the physiologic condition that activates the order and the treatment choice that follows. Three components were examined. First, we recorded the clinical trigger used to define when the CPR order applies. Trigger language was reviewed for explicit reference to absence of pulse, absence of breathing, cardiopulmonary arrest, or other physiologic conditions. Terms such as unresponsive were not treated as equivalent to pulseless or not breathing because unresponsiveness alone cannot establish cardiopulmonary arrest. Second, we recorded wording used for affirmative and negative resuscitation choices, including formulations such as Attempt Resuscitation, Attempt CPR, Do Not Attempt Resuscitation, Do Not Resuscitate, No CPR, and Allow Natural Death. Third, we assessed whether the meaning of the CPR decision was self-contained or depended on additional instructions, cross-references, abbreviations, or adjacent sections. The purpose was not to determine whether one terminology was preferable to another, but to characterize the degree to which responders encountering different jurisdictional forms would be required to translate among differing clinical triggers, labels, and decision structures.

Act assesses whether and how the CPR field communicates the response expected after the selected order is identified and understood. We extracted responder-directed language attached to affirmative and negative CPR choices and any explicit instruction describing what the responder should do after identifying the selected order. Affirmative language included instructions directing initiation or provision of resuscitative treatment. Negative language included both prohibitions against initiating resuscitation and any affirmative instructions describing the clinical course or care to be provided when resuscitation was not attempted. Action phrases were summarized descriptively and normalized to common concepts for graphical display in Figure 3.

**Figure 3:**
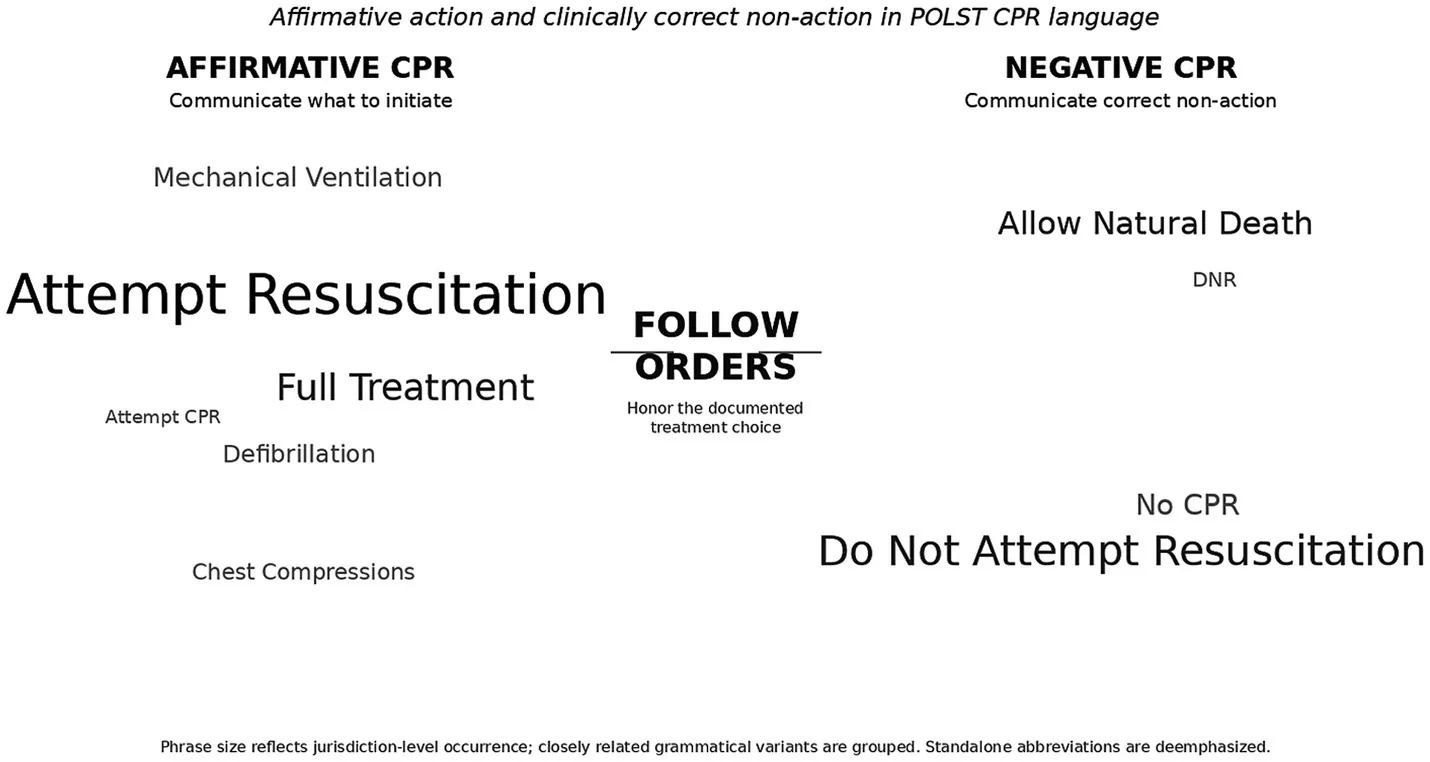
Responder action phrases. Affirmative and negative CPR language are displayed separately to show the different communication work of initiating resuscitation and communicating clinically correct non-action. Phrase size reflects jurisdiction-level occurrence; closely related grammatical variants are grouped. “Follow Orders” is shown as a bridging instruction.

Figure 3 was developed as a frequency-weighted phrase display of actions associated with affirmative and negative CPR choices. Phrase frequencies were calculated as the number of jurisdictional forms containing each phrase or grouped concept. Phrase size in the display was scaled to jurisdiction-level frequency. Closely related grammatical variants were combined before frequencies were calculated, and repeated use of the same phrase within a single form did not increase its count. Wording was retained as closely as possible to the forms. Standalone abbreviations were deemphasized when they did not independently specify responder action. “Follow Orders” was retained as a bridging instruction because it could support either CPR choice by directing the responder to act in accordance with the documented medical order.

## Discussion

This analysis examines POLST forms as communication documents intended to support recognition, interpretation, and implementation of treatment orders. Because POLST is constrained to a single page and CPR is a time-sensitive decision, the analysis focuses on the CPR decision field and the information encountered before or with it.

The *Find, Understand, Act* is a framework that is newly defined in this study. It is a variation on the generic approach to logic model development. As such, it will require more evaluation and refinement in future applications. The study is also limited because it is primarily a descriptive communication analysis rather than an assessment of legal validity, clinical effectiveness, responder performance, or patient outcomes. Measurements describe features of the forms but do not demonstrate that one design is easier to use or produces better decisions in emergencies. Jurisdictions were therefore not ranked, and observed differences should not be interpreted as measures of form quality. The analysis was restricted to the CPR field and adjacent upper-field content. Simulation or field-based research would be required to determine how the observed differences affect responder recognition, interpretation, and action under time pressure.

Despite these limitations, several features strengthen the analysis. A common measurement framework was applied across jurisdictional forms, with repeated measurement and adjudication was used to assess the visual area allocated to CPR. Visual prominence and textual density were treated as distinct properties, allowing forms to be compared without assuming that any one layout was optimal. Quantitative visual measures were also paired with examination of the clinical trigger, treatment-choice wording, and responder-directed language. Font size, text spacing, and related typographic features were not systematically measured because variation across forms did not support a consistent approach.

The *Find–Understand–Act* framework is a logic model guiding interpretation of our findings. *Find* frames the work of a responder readily locating the CPR decision within surrounding information. The analysis showed that CPR prominence and textual density were not interchangeable: forms could allocate substantial space to CPR yet contain relatively little text or provide less space while presenting greater textual burden. This distinction is particularly relevant to portability because responders may encounter forms from jurisdictions other than their own.

*Understand* begins after the decision box has been found. The responder must identify the physiologic condition that activates the order and correctly associate that condition with the authorized choice. Most forms converged on a common clinical state. There was considerable variation in terminology, labels, and the degree to which interpretation depended on cross-references, adjacent sections, or additional descriptors. The communication task therefore extends beyond locating a prominent Yes/No choice to interpret the relationship between clinical trigger and treatment instruction.

*Act* extends the framework from interpretation to implementation. For an affirmative CPR order, action language generally directs initiation of a familiar resuscitative sequence. Negative CPR orders only communicate that resuscitation should not be initiated. The frequency-weighted phrase display showed that affirmative phrases commonly named related treatment actions, whereas negative phrases were dominated by prohibitions against resuscitation. There was comparatively little language describing affirmative care preceding the No CPR decision. This asymmetry constitutes an action-language gap. This gap hints at health literacy issues for the comfort care envisioned by both health care professionals and people selecting No CPR.

The distinction matters because No CPR does not mean no care. The physiologic trigger specifying a No Resuscitation order represents the end of a process. No CPR is triggered when the heart has stopped and the person is not breathing. The decision is made prior to the event. Once a decision is made not to resuscitate, other clinical needs remain and are a part of the dying process. Active dying may include irregular breathing, mottling, deep unresponsiveness, low urine output, and death rattle. Comfort-focused care represents an affirmative clinical response to these symptoms rather than an absence of intervention (4). This is an important gap identified during this analysis.

The *Find–Understand–Act* framework therefore identifies a communication boundary that warrants further study. POLST forms generally make the resuscitation choice visible and distinguish between attempting and not attempting CPR. The findings indicate that Act language for No CPR needs to communicate care targeting the comfort needs of persons at the end of their lives who have elected not to receive resuscitation.

## Data Availability

The original contributions presented in the study are included in the article/Supplementary material, further inquiries can be directed to the corresponding author.
